# Do proxies reflect patients' health concerns about urinary incontinence and gait problems?

**DOI:** 10.1186/1477-7525-3-75

**Published:** 2005-11-23

**Authors:** Takahiro Higashi, Ron D Hays, Julie A Brown, Caren J Kamberg, Chau Pham, David B Reuben, Paul G Shekelle, David H Solomon, Roy T Young, Carol P Roth, John T Chang, Catherine H MacLean, Neil S Wenger

**Affiliations:** 1 Department of Epidemiology and Healthcare Research, Kyoto University, Yoshida-Konoe-Cho Sakyo-ku, Kyoto, 606-8501, Japan; 2 UCLA Division of General Internal Medicine and Health Services Research: 911 Broxton Plaza 3^rd ^Floor, Los Angeles, CA, 90095, USA; 3 RAND Santa Monica: 1776 Main Street P.O. Box 2138, Santa Monica, CA, 90407-2138, USA; 4 RAND Washington D.C.: 1200 South Hayes Street, Arlington VA 22202-5050, USA; 5 UCLA Division of Geriatrics: 10945 Le Conte Avenue Suite 2339, Los Angeles, CA 90095, USA; 6 Greater Los Angeles VA Healthcare System: 11301 Wilshire Boulevard, Los Angeles, CA 90073, USA

**Keywords:** Fear of falling, Urinary incontinence, Health-related quality of life, Patient-proxy agreement

## Abstract

**Background:**

While falls and urinary incontinence are prevalent among older patients, who sometimes rely on proxies to provide their health information, the validity of proxy reports of concern about falls and urinary incontinence remains unknown.

**Methods:**

Telephone interviews with 43 consecutive patients with falls or fear of falling and/or bothersome urinary incontinence and their proxies chosen by patients as most knowledgeable about their health. The questionnaire included items derived from the Medical Outcomes Study Short Form 12 (SF-12), a scale assessing concerns about urinary incontinence (UI), and a measure of fear of falling, the Falls Efficacy Scale (FES). Scores were estimated using items asking the proxy perspective (6 items from the SF-12, 10 items from a UI scale, and all 10 FES items). Proxy and patient scores were compared using intraclass correlation coefficients (ICC, one-way model). Variables associated with absolute agreement between patients and proxies were explored.

**Results:**

Patients had a mean age of 81 years (range 75–93) and 67% were female while proxies had a mean age of 70 (range 42–87) and 49% were female. ICCs were 0.63 for the SF-12, 0.52 for the UI scale, and 0.29 for the FES. Proxies tended to understate patients' general health and incontinence concern, but overstate patients' concern about falling. Proxies who lived with patients and those who more often see patients more closely reflected patient FES scores compared to those who lived apart or those who saw patients less often. Internal consistency reliability of proxy responses was 0.62 for the SF-12, 0.86 for the I-QOL, and 0.93 for the FES. In addition, construct validity of the proxy FES scale was supported by greater proxy-perceived fear of falling for patients who received medical care after a fall during the past 12 months (p < .05).

**Conclusion:**

Caution should be exercised when using proxies as a source of information about older patients' health perceptions. Questions asking about proxies' views yield suboptimal agreement with patient responses. However, proxy scales of UI and fall concern are internally consistent and may provide valid independent information.

## Background

In addition to traditional objective measures of morbidity and mortality, self-reports are increasingly used to characterize patients' health and as an outcome of medical therapy. However, patients sometimes are unable to provide information because of cognitive impairment or other communication disabilities (e.g., hearing problems and/or language incompatibilities), or severity of illness. In such cases, investigators must decide whether to substitute the missing information with a proxy responder. This decision depends, at least in part, on the validity of proxy reports, usually conceptualized as how accurately the proxy reflects the information that would have been provided by the patient. Particularly in older populations, which are more likely to have cognitive impairment, studies have addressed this issue by comparing information provided independently by patients and proxies [1-9]. In general, these studies show good agreement between patients and proxies concerning observable behavior (such as physical function), but levels of concordance tend to be lower for internal perceptions such as energy level or emotional well-being [10].

Urinary incontinence and gait problems are prevalent among older persons. Studies show that up to one third of older individuals have at least occasional urinary incontinence [11]. Persons with urinary incontinence are twice as likely to report feeling depressed as their continent counterparts [12]. Similarly, nearly one-third of community-dwelling older persons fall each year, and up to half report a "fear of falling" [13,14]. Falls may lead to serious injury such as hip fracture, and fear of falling is associated with worse mental health and physical function [14,15].

Whether proxy information can be used to estimate fear of falling and incontinence in older patients is important for research and to improve care for these conditions. Measurement of intervention effects using self-report data would lead to the exclusion of substantial proportions of patients with these conditions who are unable to provide these data. On the other hand, noise or bias is introduced into measurement if proxy responses do not accurately reflect the perspective of patients. As part of a quality improvement intervention focused on care for falls and incontinence for older patients, we evaluated the validity of proxy responses assessing patient perceptions of health, fear of falling and incontinence. We also identified variables that were significantly associated with patient-proxy agreement.

## Methods

### Sample

This study examined proxy-reported measures that could supplement patient reports in the evaluation of an intervention to improve the quality of outpatient care for urinary incontinence, falls and gait impairment, and cognitive impairment. Details of this controlled trial are described elsewhere [16]. Data presented in this report are from the enrollment phase, before any intervention. Consecutive community-dwelling patients age 75 years or older receiving care from two medical groups in southern California were interviewed by telephone to screen for these three conditions several days before a scheduled visit to their physician. Patients identified as having any of the three conditions were invited to participate in the practice-based quality improvement project.

Among 649 patients who consented to participate, 531 patients answered questions for themselves, and 118 proxies provided information for patients who could not provide information. For the purpose of examining the concordance in response between patients and proxies, we asked 44 consecutive patients among the 531 patients who provided self-report information (25 had urinary incontinence, 32 had falls and/or fear of falling) to name a "proxy," defined as the person most knowledgeable about his/her health. Proxies were contacted to participate in a telephone interview. Although proxies were interviewed separately, the majority of proxy interviews were conducted on the same day as the patient interview. The RAND and University of California, Los Angeles Institutional Review Boards approved the study protocol (UCLA IRB#G02-03-002, RAND-IRB#00000051).

### Measurements

We collected information via telephone interview on the SF-12 Health Survey [17], the Urinary Incontinence Quality of Life (I-QOL) scale [18], the Falls Efficacy Scale (FES) [14], and a proxy urinary incontinence (pUI) survey (described below). Briefly, the SF-12 is an abridged version of the Short Form-36 Health Survey [17,19,20] that measures 8 domains of health. Although the SF-12 is only one third the length of the SF-36, it accounts for more than 90% of the variance in the SF-36 physical and mental health summary scores in the U.S. population [17]. The SF-12 physical component and mental component scores (summary measures) are standardized to the U.S. normative population with a mean of 50 and standard deviation of 10. A higher score means better health. The I-QOL scale assesses patients' concerns about urinary incontinence using 22 items covering 3 domains of concern about urinary incontinence: "avoidance and limiting behavior," "psychosocial impacts," and "social embarrassment." Each item specifies patients' concern or limitation of activities due to urinary incontinence. Patients rate these items with 5 response options from "extremely" true to "not at all" true. The score of the I-QOL is the sum of the item responses converted to a 0–100 possible range with a higher score signifying *higher *quality of life. The FES assesses fear of falling during daily activities in older persons [14]. It consists of 10 items with 4 possible responses ranging from "not concerned at all" to "very concerned." Scores are computed as the sum of item responses, ranging from 10 to 40. Although the original scale was created so that a larger number indicated greater concern, we reversed the scale to make it consistent with the SF-12 and I-QOL scales. Therefore, a higher FES score signifies less concern about falling.

Because the aim of this study was to compare patient and proxy responses, we conducted separate telephone interviews with patients and proxies. After examination of the feasibility of administration, we selected for proxy administration a subset of items from the SF-12 (4 items) and created a new proxy urinary incontinence (pUI) scale from 10 items modified from the I-QOL scale. Eight items from the SF-12 and 12 items from the I-QOL scales were excluded because they were judged to measure internal perceptions, for which the literature shows poor agreement between proxy and patient responses [10], and we predicted that it would be difficult to obtain a proxy's view on these issues. Two additional physical function items from the SF-36 (capability of vigorous activities and difficulty in bathing or dressing) not contained in the SF-12 also were asked in both patient and proxy interviews. The items contained in the proxy interview are listed in Table 1. All items in the FES were judged as feasible for proxy interview. For survey items that assessed a patient's concern about urinary incontinence and falling, we asked the proxy's concern about patients rather than querying the proxy's opinion of the patient's concern to avoid confusing responding proxies.

**Table 1:** Item Descriptor, Number of Patient-Proxy Pairs and Intraclass-Correlation Coefficients of Individual Items in the pSF-12p, pUI Scale, and FES

**Item descriptor**	**N**	ICC*	**%Bias†**
pSF-12 PCS Items			

Self-report health (poor/fair/good/very good/excellent)	41	0.38	8.5%
Limitation in vigorous activities?	43	0.27	3.5%
Limitation moderate activities?	42	0.54	9.5%
Limitation in climbing several flights of stairs	43	0.39	8.1%
Limitation in dressing/bathing yourself	43	0.08	9.3%
Limited kind of activities/work	43	0.09	-16.3%

pUI Scale Items^‡^			

Worry about getting to the toilet on time	24	0.64	-8.3%
Have to be careful about sitting/standing	23	0.60	-1.1%
Worry where the toilets are in new places	24	0.51	0.0%
Don't feel free to leave home	24	0.14	-3.1%
Worry about others smelling urine on me	24	0.39	-9.4%
Frequent trip to toilet is important	24	0.43	-5.2%
Plan details in advance	24	0.29	-8.3%
Hard to get good sleep	24	0.63	-4.2%
Watch what/how much to drink	24	0.07	-4.2%
Limited choice of clothing	22	-0.10	1.1%

FES Items^‡^			

Cleaning the house	32	0.10	17.7%
Getting dressed	32	0.23	9.4%
Preparing simple meals	32	0.38	-3.1%
Taking a bath/shower	32	0.26	3.1%
Simple shopping	30	0.10	15.6%
Getting in and out of a chair	32	0.23	6.3%
Going up/down stairs	32	0.02	24.0%
Walking around the neighborhood	32	0.10	18.8%
Reaching into cabinets or closets	32	0.19	0.0%
Going to answer the telephone	32	0.09	8.3%

* Intraclass correlation

† * Mean proxy answers compared to patient answers on the item expressed as the percentage of the full scale points. Negative values indicate proxies indicated worse health on pSF and less concerned on FES and pUI.

‡ For proxy interview, the pUI and FES items were modified to address proxy's concerns about patient UI and fear of patient falling

Since only subsets of items were used in proxy interviews for the SF-12 PCS and I-QOL scales, we predicted full-item scores for these scales (termed pSF-12 PCS and pUI, respectively) using a weighted combination of items. Weights for items in the pSF-12 PCS and pUI were obtained from the coefficients from linear regression models with dependent variables of SF-12/I-QOL scores calculated from the full set of items to which patient responded, and the predictor variables of *patient *responses to items included in proxy interviews (6 items for SF-12 and 10 items for I-QOL). Coefficients used to compute pSF-12 PCS and pUI scales are available from the first author. The regression models were fitted using responses from the 531 interviewed patients. For the regression, we performed a complete case analysis excluding cases with any missing items (SF-12: N = 488, I-QOL: N = 179). The estimated SF-12 PCS and I-QOL scores accounted for 85% and 92% of the variance, respectively, of the scores calculated by the full set of items. We did not calculate SF-12 mental component scores because published information indicates that proxies do not provide valid information about patients' mental health.

Five proxies did not answer one or two items (2 proxies for pSF-12, 1 for pUI scale, 1 for the FES and 1 for both the pUI scale and the FES). We computed alternative coefficients for the available items based on regression models without the missing variables. One patient failed to answer one item of the pSF-12 PCS, so the score for this patient was estimated from a separate regression model using 11 available items and 2 extra SF items. For the I-QOL scale and the FES, missing items were imputed from the mean of the other item responses when the number of missing items was 3 or fewer for I-QOL and 2 or fewer for FES. One proxy was excluded from the analysis because he did not answer more than half of the scale items. For the purpose of comparison, the predicted scores from the regression model (i.e., pSF-12, pUI scores rather than original SF-12, or I-QOL scores) were used for both patient and proxy.

Additionally, a self-administered survey was mailed to proxies asking about their own age, education level, whether or not they lived with the patient, how often they saw the patient, how often they accompanied the patient to physician visits, and their level of knowledge about various aspects of the patient's health, medication, mood and activities.

### Statistical analysis

Proxy scores on the pSF-12 PCS, pUI, and FES scales were compared with patient response scores using intraclass correlation coefficients (ICC, one-way model) that represent both correlations and systematic mean differences [21]. In order to evaluate the relationship between proxy characteristics and the agreement of proxy-patient responses, the absolute difference in scores was computed. The absolute difference represents the discrepancy between the proxy and patient reponses ignoring the direction of the difference. Since score ranges varied across scales, the absolute difference was also expressed in terms of the patient score standard deviation as well as the percentage of each scale range. We used one-way ANOVA to evaluate mean absolute difference in scores across groups of proxies if the assumption of equal variance held; otherwise the Kruskal-Wallis test was used. The assumption of equal variance was considered to be violated when Bartlett's test rejected the null hypotheses at the level of p = 0.10. Otherwise, the statistical signficance level was set at 0.050.

We used the full 118 proxies from the intervention study for the purpose of examining internal consistency reliability of the pSF-12 PCS, pUI and the FESusing Cronbach's alpha [22]. For the calculation of alpha, item responses were assigned the weights (available from the first author) used in the score calculation for the pSF-12 PCS and pUI. In addition, construct validity of the proxy response FES scale was assessed. For FES, the proxies for patients were divided into 3 groups of ascending fall severity: 1) those who had not fallen in the past 12 months, 2) those who had fallen but did not need to see a provider, and 3) those who had fallen and needed to see a provider. Scores across these groups were tested using the Cuzick's test for trend [23]. Statistical analysis was performed using STATA version 8.2 (STATA Corporation, College Station, TX).

## Results

### Patient and proxy characteristics

The 43 patients in this analysis had a mean age of 81 (range 75–93) and 29 (67%) were female. Proxies had a mean age of 70 (range 42–87) and 21 (49%) were female. Proxies were related to patients as follows: 16 husbands (37%), 11 daughters (26%), 7 wives (16%), 4 sons (9%), and 5 others (2 roommates, 1 sister, 1 fiancé and 1 niece). Thirty-five proxies lived with the patients. One of the 43 proxies who completed a telephone interview did not return the mail survey and was excluded from the analysis of the association between proxy characteristics and agreement. A summary of the scores for the SF-12 PCS, FES, pUI and pSF-12 PCS for the patients and proxies in this study, as well as all patients and proxies in the intervention study sample, are presented in Table 2. The scores on these scales for the 43 patients in this substudy were not different from the full patient intervention study sample.

**Table 2:** Mean Scores for Study Sample and the Full Intervention Project Patient Sample

	**Dyad study sample**					
	**Patient**			**Proxy**		
	
	Mean	SD^#^	N	Mean	SD^#^	N
SF-12 PCS*	37.4	(11.5)	43			
pSF-12 PCS^†^	37.0	(9.9)	43	36.2	(9.2)	43
I-QOL^‡^	78.1	(17.8)	24			
pUI Scale^§^	76.5	(18.4)	24	80.9	(20.0)	24
FES^¶^	31.8	(7.2)	32	28.8	(7.0)	32

	**Full intervention project sample**					
	**Patient**			**Proxy**		
	
	Mean	SD^#^	N	Mean	SD^#^	N

SF-12 PCS*	36.7	(10.7)	492			
pSF-12 PCS^†^	36.6	(9.8)	509	31.8	(9.7)	117
I-QOL^‡^	73.4	(20.4)	203			
pUI Scale^§^	73.4	(19.6)	198	50.7	(23.6)	26
FES^¶^	30.4	(7.8)	411	21.6	(9.2)	72

*SF-12 PCS: Short Form 12 physical component score †pSF-12: proxy version of SF-12

‡I-QOL: Incontinence quality of life scale §pUI: proxy urinary incontinence, ¶FES: falls efficacy scale, #SD: standard deviation.

Note: Number of patients that had SF-12 and pSF-12 scales are different because some patients had missing values.

### Agreement between patients and proxies

**Figure 1:**
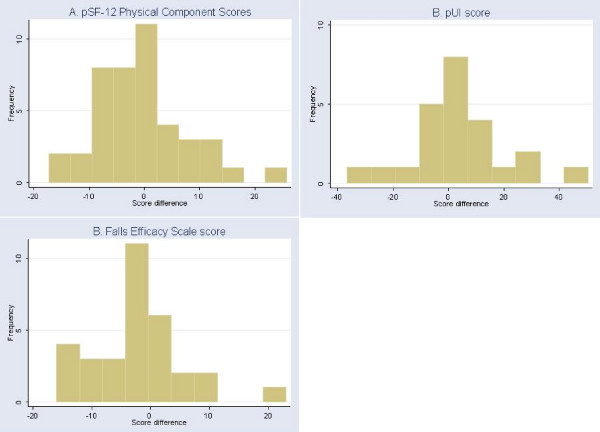
**Differences in estimated SF-12 PCS, pUI, and FES scores between proxies and patients (proxy score) – (patient score)**. * >0 indicates proxy score is greater than patient score.

Intraclass correlations between patients and proxies on the predicted scores were 0.63 for pSF-12 PCS, 0.52 for pUI scores, and 0.29 for the FES scores. The distribution of the differences between patient scores and proxy scores are illustrated in Figure 1. On average, proxy scores were lower than patient scores by 0.8 for the pSF-12 PCS (i.e., proxies underestimate patients' health status), 3.0 points lower on the 40-point FES (i.e., proxies are more concerned than patients about patients falling), and 4.4 higher on the 0–100 pUI scores (i.e., proxies are less concerned about incontinence than patients). The mean *absolute *difference in scores between patients and proxies was 6.2 for the pSF-12 (0.6 standard deviation (SD)), 13.0 for the pUI (0.7 SD) and 6.2 for the FES (0.9 SD). Ninety percent of proxy scores fell within 14 absolute difference points for pSF-12 scores (1.4 SD), 32 points for pUI scores (1.7 SD), and 15 points for FES scores (2.1 SD). One proxy, a patient's fiancé, provided answers to FES items that were extremely discrepant from the patient's answers, resulting in an FES score difference of 23. Excluding this outlier pair, the ICC, mean raw and absolute difference between patient score and proxy score (patient score minus proxy score) for the FES was 0.40, 3.8 (i.e., proxies are more concerned), and 5.6 (19% of possible score range, 0.8 SD), respectively.

Table 3 shows the relationship of the mean absolute difference of each scale to proxy characteristics. Proxy characteristics were generally unrelated to agreement with patient reported health. However, proxies who lived with patients and those who saw patients every day had a significantly smaller mean absolute difference from patient scores on the FES than those who lived apart or those who saw patients less frequently. Proxy reports of familiarity with patients' medications, activity and mood were unrelated to agreement with patient reports on any scale. Similarly, proxy educational level and relationship with the patient did not predict accuracy concerning physical health or incontinence or falls concerns.

**Table 3:** Mean Absolute Difference between Patient and Proxy Responses for pSF-12 Physical Component Score(PCS), pUI scale, and FES Scores

		**pSF-12 PCS**			**pUI scale**			**FES**		
		
**Questions**		**N**	**mean absolute difference**	**(P value)**	**N**	**mean absolute difference**	**(P value)**	**N**	**mean absolute difference**	**(P value)**
Relationship of proxy to patient	Husband	16	5.4		12	11.9		10	5.4	
	Wife	7	9.8		2	4.3		6	5.3	
	Son	4	5.8		2	30.2		4	0.9	
	Daughter	11	4.8		5	5.3		8	8.7	
	Others+	5	7.0	(0.32)	3	24.7	(0.16)*	4	9.8	(0.14)*
Proxy's gender	Male	21	5.7		14	14.5		15	4.1	
	Female	22	6.2	(0.52)	10	10.9	(0.52)	17	8.0	(0.06)*
Proxy's educational level	Professional	7	7.0		4	10.7		4	8.0	
	College	14	5.5		8	16.6		13	7.4	
	Vocational	14	6.0		7	15.2		10	4.4	
	High School	7	7.2	(0.87)	4	7.6	(0.70)	5	5.2	(0.60)
Proxy lives with patient?	Yes	35	6.6		21	11.5		25	4.8	
	No	8	4.4	(0.28)	3	24.0	(0.12)	7	11.0	(0.05)*
How often proxy accompanies patient to MD visit?	Always	17	7.5		11	13.9		13	5.7	
	Usually	7	7.2		3	10.1		5	5.0	
	Sometimes	13	4.4		6	14.7		11	7.5	
	Never	5	5.0	(0.40)	3	13.8	(0.97)	3	5.7	(0.85)
Days per week proxy sees patient?	7 days	29	5.9		17	13.1		20	4.3	
	<7 days	9	4.7	(0.51)	3	24.0	(0.21)	8	10.1	(0.05)*
	No answer	4	11.5		3	6.0		4	7.6	
How well proxy knows about patient's health?	Very well	29	6.5		15	11.1		21	7.7	
	Pretty well	10	6.1		6	15.3		9	3.8	
	Somewhat	3	3.2	(0.59)	2	27.0	(0.26)	2	1.2	(0.08)*
Is proxy familiar with patient's meds?	Yes	36	6.3		19	13.9		27	6.6	
	No	3	4.1		2	3.6		3	1.0	
	Not sure	2	7.9		1	36.6		2	8.5	
	Not Taking	1	4.0	(0.83)	1	4.4	(0.19)	0		(0.25)
How well proxy knows about patient's activity?	Very well	36	6.2		22	14.0		27	6.7	
	Pretty well	5	5.3		1	5.0		4	4.0	
	Somewhat	1	11.2	(0.59)	0		(0.52)	1	1.0	(0.48)
How well proxy knows about patient's mood?	Very well	24	6.3		11	13.0		18	7.4	
	Pretty well	16	6.3		12	14.1		12	4.7	
	Somewhat	2	3.8	(0.81)	0		(0.84)	2	4.0	(0.42)

Note: Information on relationship to patient, gender and whether or not proxy lives with patient was available from patients, thus, includes a person who did not respond to the mail survey.

* P value derived from Kruskal-Wallis test because test for equal variance did not hold.

+ Others included roommate fiancé, niece, and sister.

Intraclass correlation coefficients of individual items ranged from -0.10 to 0.64 (Table 1). The items with poorest agreement among pSF-12 items were whether "health limits dressing/bathing" and whether "health limits kind of activities/work,"; among pUI items, whether "urinary incontinence limits choice of clothing" and whether he/she has to "watch what/how much to drink"; and among FES items, whether there is concern about falling when "going up/down stairs," "going to answer the telephone," doing "simple shopping," and "walking around the neighborhood". The best agreement was found for whether "health limits moderate activities" among pSF-12 items, "worry about getting to the toilet on time" among pUI items and concern about falling "when preparing simple meals" among FES items.

### Reliability and validity of proxy responses

Cronbach's alphas for proxy response scales were 0.62 for the pSF-12 PCS scale, 0.86 for pUI scale and 0.93 for the FES. Table 4 shows the correlation coefficients between individual items and the scale, and scale alphas when the score was calculated without each item.

**Table 4:** Correlation between Item and Scale and Cronbach's Alpha of Proxy Scales When Each Item is Deleted

	**N of proxies**	**Item-scale correlation**	Cronbach's alpha*
**pSF-12 PCS Items**			

Self-report health (poor/fair/good/very good/excellent)	118	0.57	0.58
Limitation in vigorous activities?	118	0.55	0.62
Limitation in moderate activities?	118	0.81	0.47
Limitation in climbing several flights of stairs	118	0.63	0.55
Limitation in dressing yourself	118	0.26	0.64
Limited kind of activities/work	117	0.79	0.54
			Overall = 0.62

**pUI Scale Items**			

Worry about getting to toilet on time	28	0.72	0.84
Have to be careful about sitting/standing	27	0.77	0.84
Worry where the toilets are in new places	28	0.72	0.84
Don't feel free to leave home	27	0.69	0.84
Worry about others smelling urine on me	28	0.84	0.83
Frequent trip to toilet is important	28	0.67	0.85
Plan details in advance	28	0.70	0.85
Hard to get good sleep	27	0.43	0.87
Watch what/how much to drink	28	0.54	0.86
Limited choice of clothing	28	0.72	0.85
			Overall = 0.86

**FES Items**			

Cleaning the house	69	0.79	0.93
Getting dressed	81	0.83	0.92
Preparing simple meals	68	0.84	0.92
Taking a bath/shower	79	0.83	0.93
Simple shopping	62	0.84	0.93
Getting in and out of a chair	81	0.79	0.93
Going up/down stairs	74	0.69	0.93
Walking around the neighborhood	72	0.82	0.93
Reaching into cabinets or closets	76	0.75	0.93
Going to answer the telephone	71	0.77	0.93
			Overall = 0.93

* Cronbach's alpha of scales when each item is delete

The mean FES proxy score was 26.0 (n = 20) for the patients who hadn't fallen in the past 12 months, 21.8 for the patients who had fallen but didn't need to see a provider (n = 22), and 18.4 for patients who had fallen and needed to see a provider (n = 18). This score trend was statistically significant (p = 0.009).

## Discussion

This study demonstrated that agreement between proxies and patients is not very high for reports about physical health and concerns about incontinence and falls. Neither the pSF-12 PCS, the pUI nor the FES reached the intraclass correlation coefficient cut off of 0.7 that is generally considered acceptable agreement [24]. Directionality of difference in scores indicated that proxies tended to underestimate patient health and be more concerned about patient gait problems, but less concerned about urinary incontinence. Compared to prior literature, which generally shows that proxies overestimate functional impairment, our findings for the SF-12 and the FES are consistent, but the finding for the concern about urinary incontinence is not. This may reflect privacy concerns regarding urinary incontinence and patients' reluctance to talk about it. Alternatively, it may suggest that fear of falling is of greater concern because of the potential for injury, disability and financial costs.

Despite suboptimal agreement with patient responses, proxy responses appeared to be internally consistent and the results of this study provide support for construct validity of proxy FES, suggesting that these scales may measure proxy responses that represent important constructs that are distinct from the patient responses. Our decision to ask proxies' concern rather than proxy estimate of patients' concern may have strengthened this tendency because proxy's own concern will be less influenced by guessing. The alternative approach of asking the proxy to estimate the patient's level of concern may be more likely to produce missing values and becomes particularly problematic for patients with severely compromised cognition. However, further study should test proxy items that ask about patient concern to evaluate whether these questions better reflect patient responses.

Proxies' individual characteristics did not predict agreement between patients and proxies on these three scales. Though most proxies reported that they were knowledgeable about patients' health, mood, activities and medications, they did not well replicate patient responses, and proxy report of familiarity with these issues was unrelated to agreement. Rather than self-assessment of familiarity, structural characteristics such as whether the patient and proxy live together or how often they see each other were better associated with proxy agreement with patient scores, at least for the FES.

In our study, the comparison across proxy characteristics was made only among proxies who were considered most knowledgeable about the patients' health. A subject could have identified a proxy who was most knowledgeable about his/her health but did not live with him/her. Our finding of better prediction of agreement by structural characteristics does not necessarily mean that selecting proxies primarily based on structural characteristics will result in better proxies. Future study is necessary to explore the best strategy to select optimal proxies.

Our study has several limitations. First, patients in the analyses of agreement had intact cognitive function and were well enough to participate in the survey process so their proxies may not have needed to be familiar with patient health status and concerns, since patients could communicate this information on their own. "Real" proxies who take care of patients may provide more accurate information than the "forced" proxies used for practical reasons in this study, though this is impossible to prove. Second, we used only items from the SF-12 and I-QOL scales that appeared to be feasible for proxy interview and we approximated the proxy score by re-weighting these responses. Since we included only items that we judged to be feasible, this may overestimate the agreement compared to the entire set of items from the original scales. Finally, the sample size of this study was small and there was limited statistical power for the evaluation of proxy factors associated with agreement. Nonetheless, our findings are generally consistent with prior research regarding the effect of proxy characteristics on patient-proxy agreement: demographics such as age, gender and education are inconsistent predictors of agreement [25-29], while greater contact such as living together or frequent visits are reasonable, though not perfect, predictors of agreement [9,25,29]. Larger studies will need to identify the role of factors related to the accuracy of proxy health reports for patient subjective health.

## Conclusion

Our study shows that the agreement between patient and proxy responses for SF-12, urinary incontinence and FES scales are less than acceptable. However, there is support for the reliability and construct validity of proxy responses. Rather than replacing patient responses, proxy responses may be better used in separate analysis. Future research should evaluate other scale properties, including test-retest reliability and responsiveness to change, of proxy responses, which may, on their own, be valuable scales.

## Authors' contributions

TH, RH, DR, PG, CR, and NW contributed to concept and design, acquisition, analysis, and interpretation of data and preparation of manuscript. JB, CJ, and CP contributed to concept and design, acquisition of data and preparation of manuscript. DS and CM contributed to concept and design, analysis and interpretation of data and preparation of manuscript. RY contributed to concept and design, interpretation of data and preparation of manuscript. JC contributed to concept and design, and interpretation of data and preparation of manuscript. All authors read and approved the final manuscript.
